# Mucocele of the glands of Blandin-Nuhn: a case report

**Published:** 2013-03-30

**Authors:** Nathalia Garcia Leon, Gilberto E Marrugo Pardo

**Affiliations:** a Otorhinolaryngology Unit. Universidad Nacional de Colombia, E-mail: gemarrugop@unal.edu.co

**Keywords:** Blandin nuhn glands, Mucocele

## Abstract

Mucoceles arising from the Blandin Nuhn glands are uncommon benign lesions of the oral cavity, which by their clinical presentation may be confused with more serious diseases such as vascular lesions, pyogenic granulomas, polyps, or squamous papillomas; thereby, it is convenient to be aware of the characteristics of this entity to guide the accurate and timely diagnosis and treatment. Herein, we present a case of a 10-year-old patient with a recurrent lesion of this type, which required surgical excision and marsupialization of the same, with no evidence of recurrence during follow-up.

## Abstract (es)

Los mucoceles originados de las glándulas de Blandin Nuhn son lesiones benignas poco comunes de la cavidad oral, que por su presentación clínica pueden ser confundidas con patologías de mayor gravedad como lesiones vasculares, granulomas piógenos, pólipos o papilomas escamosos; por lo cual es conveniente conocer las características particulares de esta entidad para orientar de manera precisa y oportuna su diagnóstico y tratamiento. Se presenta el caso de una paciente de 10 años con una lesión recidivante de este tipo, que requirió de extirpación quirúrgica y marsupialización de la misma, sin evidencia de recurrencia durante el seguimiento.

## Introduction

Mucoceles are benign cystic lesions of the oral cavity, which are developed from the extravasation or retention of mucin produced by the minor salivary glands, which are found throughout the surface of the oral cavity, except in the gingival sulcus; in spite of their great distribution, prevalence depends on their location. Mucoceles of the lower lip are the most common (77.9%), followed by lingual mucoceles and mucoceles of the floor of the mouth (15.6%). Mucoceles on the ventral surface of the tongue derived from the Blandin Nuhn glands are considered quite unusual^1^, which because of their clinical characteristics and location may be confused for other pathologies like vascular lesions, pyogenic granulomas, squamous papillomas, among others.

## Clinical case

**Figure 1:**
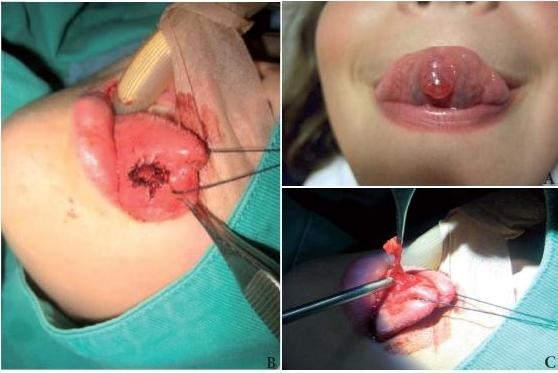
A Lesion on the ventral surface of the tongue, B and C Image of the surgical resection and marsupialization of the lesion.

Herein, we present a female 10-year-old patient who consulted for a condition with three weeks of evolution of a midline lesion of the tongue ventral surface, characterized as a cystic, sessile, reddish mass, of progressive growth, associated to occasional pain Fig. 1 (A), which six months back had been operated at another institution with diagnosis of mucocele, with later reappearance of such. Because of the recurrence, the patient was taken to surgery. Under general anesthesia, complete resection was performed of the mass with marsupialization Fig. 1 (B and C). The pathology reported changes compatible with extravasation-type mucocele of the ventral surface of the tongue, which because of its location and presentation corresponds to a mucocele of the Blandin Nuhn glands. The patient continued with out-patient follow up for 10 months without clinical evidence of recurrence.

## Discussion

The minor salivary glands are a set of three types of glands found throughout the oral cavity, except in the gingival sulcus^2^. The Von Ebner glands drain on the base of the circumvallate and foliate papillae on the dorsal lingual surface; the Weber glands, mucin exclusive producers, are located on the lateral surface of the tongue, having as drainage site the lingual tonsil crypts; lastly, the Blandin Nuhn glands are found near the tip of the ventral surface of the tongue, and are surrounded by lingual muscles near the medial line. These glands extend laterally and towards the rear of the medial line, forming a horseshoe-like mass with an aperture toward the posterior draining through four to six ducts opening near the lingual frenulum; the characteristics of their secretion products have not been precisely determined, but have been histologically described as a set of seromucous acini in their anterior part associated to mucous acini in their posterior part^3^.

The cystic formations or mucoceles of the Blandin Nuhn glands have been reported as unusual; according to Harrison^4^ in a review of 400 mucoceles of the oral cavity only nine originated in the tongue, constituting 2.3%; according to Saza *et al*.,^5^ mucoceles of the Blandin Nuhn glands were found in 9.6% of 385 mucoceles studied. In the series published by Jinbu *et al*.,^1^ mucoceles from the Blandin Nuhn glands constitute 9.9% of all oral mucoceles. Young patients seem to develop mucoceles with greater frequency than older patients; in the Brazilian case series of 104 patients reported by Nico *et al*.,^6^ 50% were younger than 20 years of age and 34.6% were under 15 years of age. A greater incidence was also found in females.

Harrison^4^ reported that in younger patients the mucoceles are extravasation types that emerge as a consequence of local bite-type traumatism causing rupture of the excretory complex with mucous extravasation. Histologically, they consist of a circumscribed cavity full of mucin between the connective tissue and the submucosa provoking an increase of the mucosa with thinning of the epithelial surface, surrounded by granulation tissue and inflammatory cells with the fundamental characteristic that they lack epithelial lining, contrary to retention mucoceles, which are real cysts constituting a cavity lined by a layer of squamous epithelium, associated to canalolitiasis and sialadenitis syndromes, most prevalent in populations over 40 years of age^7^.

The diagnosis of mucoceles of the Blandin Nuhn glands stems from their clinical characteristics, although they may resemble a vascular lesion, a pyogenic granulomas, polyps, or squamous papillomas; for this reason, it is important to raise awareness amongst the medical population of this pathology.Given their location, these types of lesions are exposed to repeated trauma with subsequent irritation and discomfort for the patient.

This is why in spite their benign characteristics, their treatment is complete surgical excision, which may or may not include marsupialization of the lesion according to its size. According to Baurmash^8^, complete extirpation of the mucocele must be guaranteed with the extraction of all the glands adjacent to the lesion or its marsupialization to avoid recurrences. Other therapeutic modalities reported are cryosurgery^9^, laser ablation^10^, and preoperative steroid injection to improve visualization of the intra-surgical lesion^11^. In this case, we performed extirpation of the lesion, marsupialization, and resection of the adjacent glands, registering good postoperative evolution without recurrence during follow up. Due to this, we recommend this approach in cases of big lesions or recurrences.
